# Tris[3,5-bis­(trifluoro­meth­yl)phen­yl]phosphine oxide

**DOI:** 10.1107/S1600536809013488

**Published:** 2009-04-22

**Authors:** Omar bin Shawkataly, Mohd. Aslam A. Pankhi, Mohamed Ismail Mohamed-Ibrahim, M. Razak Hamdan, Hoong-Kun Fun

**Affiliations:** a Chemical Sciences Programme, School of Distance Education, Universiti Sains Malaysia, 11800 USM, Penang, Malaysia; b X-Ray Crystallography Unit, School of Physics, Universiti Sains Malaysia, 11800 USM, Penang, Malaysia

## Abstract

In the title compound, C_24_H_9_F_18_OP, an intra­molecular C—H⋯O short contact generates a five-membered ring, producing an *S*(5) ring motif. The dihedral angles between the benzene rings are 57.68 (10), 77.80 (11) and 79.48 (10)°. Each of the six trifluoro­methyl substituents shows rotational disorder over two positions with refined site-occupany ratios of 0.64 (3)/0.36 (3), 0.649 (14)/0.351 (14), 0.52 (2)/0.48 (2), 0.545 (16)/0.455 (16), 0.774 (9)/0.226 (9) and 0.63 (5)/0.37 (5). The crystal structure is stabilized by inter­molecular C—H⋯O and C—H⋯F inter­actions.

## Related literature

For C—F bond lengths, see: Allen *et al.* (1987 ▶). For the stereochemistry of triphenyl­phosphine oxide complexes and for P—C bond distances, see: Bandoli *et al.* (1970 ▶); Ruban & Zabel (1976 ▶); Baures & Silverton (1990 ▶); Lynch *et al.* (1992 ▶); Shawkataly *et al.* (1997 ▶). For hydrogen-bond motifs, see: Bernstein *et al.* (1995 ▶).
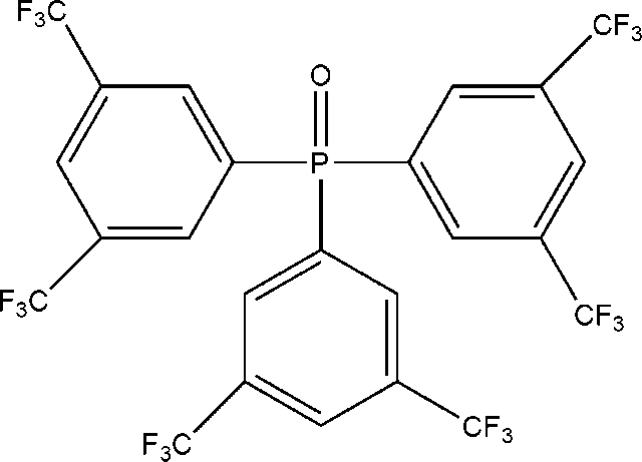


## Experimental

### 

#### Crystal data

C_24_H_9_F_18_OP
*M* *_r_* = 686.28
Triclinic, 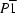

*a* = 10.7937 (2) Å
*b* = 11.8786 (10) Å
*c* = 12.5066 (10) Å
α = 111.065 (10)°
β = 103.645 (10)°
γ = 106.562 (10)°
*V* = 1327.7 (3) Å^3^
*Z* = 2
Mo *K*α radiation
μ = 0.25 mm^−1^
*T* = 294 K
0.48 × 0.38 × 0.22 mm

#### Data collection

Bruker SMART APEXII CCD area-detector diffractometer
Absorption correction: multi-scan (**SADABS**; Bruker, 2005 ▶) *T*_min_ = 0.890, *T*_max_ = 0.947
7134 measured reflections
4535 independent reflections
3611 reflections with *I* > 2σ(*I*)
*R*_int_ = 0.021

#### Refinement

*R*[*F* ^2^ > 2σ(*F* ^2^)] = 0.041
*wR*(*F* ^2^) = 0.113
*S* = 1.02
4535 reflections
565 parameters
168 restraints
H-atom parameters constrained
Δρ_max_ = 0.20 e Å^−3^
Δρ_min_ = −0.27 e Å^−3^

### 

Data collection: *APEX2* (Bruker, 2005 ▶); cell refinement: *SAINT* (Bruker, 2005 ▶); data reduction: *SAINT*; program(s) used to solve structure: *SHELXTL* (Sheldrick, 2008 ▶); program(s) used to refine structure: *SHELXTL*; molecular graphics: *SHELXTL*; software used to prepare material for publication: *SHELXTL* and *PLATON* (Spek, 2009 ▶).

## Supplementary Material

Crystal structure: contains datablocks global, I. DOI: 10.1107/S1600536809013488/sj2588sup1.cif

Structure factors: contains datablocks I. DOI: 10.1107/S1600536809013488/sj2588Isup2.hkl

Additional supplementary materials:  crystallographic information; 3D view; checkCIF report

## Figures and Tables

**Table 1:** Hydrogen-bond geometry (Å, °)

*D*—H⋯*A*	*D*—H	H⋯*A*	*D*⋯*A*	*D*—H⋯*A*
C18—H18*A*⋯O1	0.93	2.58	2.992 (3)	108
C10—H10*A*⋯O1^i^	0.93	2.38	3.203 (3)	147
C20—H20*A*⋯F10*A*^ii^	0.93	2.50	3.418 (12)	167

Symmetry codes: (i) 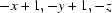; (ii) 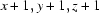.

**Fig. 1.**
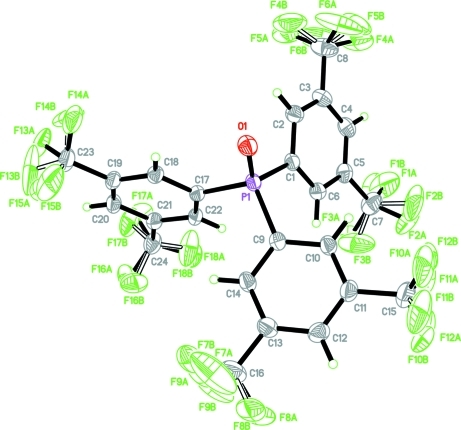
The molecular structure of the title compound, showing 50% probability displacement ellipsoids and the atomic numbering scheme. Open bonds link the F atoms of the minor disorder components.

## supplementary crystallographic information

### Comment

There has been considerable research on the stereochemistry of triphenyl phosphine oxide complexes (Bandoli *et al.,* 1970; Ruban & Zabel, 1976; Spek, 1987; Baures & Silverton, 1990; Shawkataly *et al.,* 1997) involving different space groups. A search of the Cambridge Structural Database (Version 5.29; Allen, 2007) revealed only 9 reported structures of the parent triphenylphosphine complexes as opposed to complexes of triphenylphosphine oxides. Our interest in complexes of triphenylphosphine led us to determine the *X*-ray crystal structure of the title compound, Fig. 1, in order to elucidate its conformation.

The bond lengths and angles of the title compound, Fig. 1, are within normal ranges (Allen *et al.,* 1987). An intramolecular C—H···O hydrogen bond generates a five-membered ring, producing a *S(5)* ring motif (Bernstein *et al.,* 1995). The P atom binds to three C atoms and one O atom in a nearly ideal tetrahedral geometry with the average O—P—C bond angle 112.84°. In the title compound, the P═O distance of 1.475 (3)Å, is less than the value reported for triphenylphospine oxide [1.487 (2)Å (Baures & Silverton, 1990)]. The average P—C bond distance is 1.813Å which is slightly longer than the distance observed in triphenylphosphine oxides previously studied; 1.799 (3)Å in OPPh_3_ (Baures & Silverton, 1990) and 1.76 (1)Å in its adduct with tricarboxylic acid (Lynch *et al.,* 1992). This slight lengthening is probably due to the presence of electron withdrawing -CF_3_ groups bonded to the phenyl rings. The trifluoromethyl substituents show rotational disorder over two positions with a refined site-occupany ratio of 0.64 (3)/0.36 (3), 0.649 (14)/0.351 (14), 0.52 (2)/0.48 (2), 0.545 (16)/0.455 (16), 0.774 (9)/0.226 (9), and 0.63 (5)/0.37 (5) and average C—F distance of 1.300Å. This value is slightly shorter than that of the normal -CF_3_ bond distance (Allen *et al.,* 1987).

The mean C—P—C bond angle is 105.90°, while the O—P—C bond angles show a slight variation: 113.01 (10), 114.31 (9) and 111.18 (10)° for O—P—C9, O—P—C1, and O—P—C17 respectively. The dihedral angles between the phenyl rings and the planes containing O, P and the corresponding *ipso*-C atoms are 79.52 (13), 29.14 (13), 10.70 (13)°, respectively. These are close to the values for the unsubstituted analogue [77.2 (1), 36.3 (1) and 11.9 (1)°]. The crystal structure is stabilized by intermolecular C—H···O and C—H···F interactions (Table 1).

### Experimental

The title compound was supplied by Strem Chemicals. Single crystals of (I) were obtained by slow evaporation of an ethanol solution.

### Refinement

All of the hydrogen atoms were positioned geometrically and refined with a riding approximation model with C—H = 0.93 Å and U_iso_(H) = 1.2 U_eq_(C). The trifluoromethyl substituents show rotational disorder over two positions with a refined site-occupany ratios of 0.64 (3)/0.36 (3) for F1, F2, F3, 0.649 (14)/0.351 (14) for F4, F5, F6, 0.52 (2)/0.48 (2) for F7, F8, F9, 0.545 (16)/0.455 (16) for F10, F11, F12, 0.774 (9)/0.226 (9) for F13, F14, F15 and 0.63 (5)/0.37 (5) for F16, F17, F18 respectively. Rigid-bond restraints were applied for the fluorine groups in order to improve the high displacement ellipsoids of the groups but this was not particularly succesful, except that the refinement convergence was more stable.

### Crystal data

**Table:**

C_24_H_9_F_18_OP	*Z* = 2
*M**_r_* = 686.28	*F*(000) = 676
Triclinic, *P*1	*D*_x_ = 1.717 Mg m^−^^3^
Hall symbol: -P 1	Mo *K*α radiation, λ = 0.71073 Å
*a* = 10.7937 (2) Å	Cell parameters from 5230 reflections
*b* = 11.8786 (10) Å	θ = 1.9–28.3°
*c* = 12.5066 (10) Å	µ = 0.25 mm^−^^1^
α = 111.065 (10)°	*T* = 294 K
β = 103.645 (10)°	Block, colourless
γ = 106.562 (10)°	0.48 × 0.38 × 0.22 mm
*V* = 1327.7 (3) Å^3^	

### Data collection

**Table:**

Bruker SMART APEXII CCD area-detector diffractometer	4535 independent reflections
Radiation source: fine-focus sealed tube	3611 reflections with *I* > 2σ(*I*)
graphite	*R*_int_ = 0.021
φ and ω scans	θ_max_ = 25.0°, θ_min_ = 1.9°
Absorption correction: multi-scan (*SADABS*; Bruker, 2005)	*h* = −11→12
*T*_min_ = 0.890, *T*_max_ = 0.947	*k* = −13→14
7134 measured reflections	*l* = −14→10

### Refinement

**Table:**

Refinement on *F*^2^	Primary atom site location: structure-invariant direct methods
Least-squares matrix: full	Secondary atom site location: difference Fourier map
*R*[*F*^2^ > 2σ(*F*^2^)] = 0.041	Hydrogen site location: inferred from neighbouring sites
*wR*(*F*^2^) = 0.113	H-atom parameters constrained
*S* = 1.01	*w* = 1/[σ^2^(*F*_o_^2^) + (0.0656*P*)^2^ + 0.1383*P*] where *P* = (*F*_o_^2^ + 2*F*_c_^2^)/3
4535 reflections	(Δ/σ)_max_ = 0.001
565 parameters	Δρ_max_ = 0.20 e Å^−^^3^
168 restraints	Δρ_min_ = −0.27 e Å^−^^3^

### Special details

**Table:**

Geometry. All esds (except the esd in the dihedral angle between two l.s. planes) are estimated using the full covariance matrix. The cell esds are taken into account individually in the estimation of esds in distances, angles and torsion angles; correlations between esds in cell parameters are only used when they are defined by crystal symmetry. An approximate (isotropic) treatment of cell esds is used for estimating esds involving l.s. planes.
Refinement. Refinement of F^2^ against ALL reflections. The weighted R-factor wR and goodness of fit S are based on F^2^, conventional R-factors R are based on F, with F set to zero for negative F^2^. The threshold expression of F^2^ > 2sigma(F^2^) is used only for calculating R-factors(gt) etc. and is not relevant to the choice of reflections for refinement. R-factors based on F^2^ are statistically about twice as large as those based on F, and R- factors based on ALL data will be even larger.

### Fractional atomic coordinates and isotropic or equivalent isotropic displacement parameters (Å^2^)

**Table:**

	*x*	*y*	*z*	*U*_iso_*/*U*_eq_	Occ. (<1)
P1	0.69584 (5)	0.56123 (5)	0.16458 (4)	0.03853 (15)	
O1	0.66832 (14)	0.62576 (14)	0.08623 (14)	0.0516 (4)	
C1	0.56380 (18)	0.52657 (18)	0.22911 (18)	0.0408 (4)	
C2	0.4957 (2)	0.6106 (2)	0.24927 (19)	0.0473 (5)	
H2A	0.5191	0.6809	0.2305	0.057*	
C3	0.3928 (2)	0.5897 (2)	0.2974 (2)	0.0532 (5)	
C4	0.3564 (2)	0.4856 (2)	0.3248 (2)	0.0552 (5)	
H4A	0.2866	0.4716	0.3562	0.066*	
C5	0.4241 (2)	0.4021 (2)	0.3053 (2)	0.0506 (5)	
C6	0.5283 (2)	0.42242 (19)	0.25787 (19)	0.0460 (5)	
H6A	0.5740	0.3661	0.2455	0.055*	
C7	0.3816 (3)	0.2847 (3)	0.3293 (3)	0.0733 (7)	
C8	0.3197 (4)	0.6811 (4)	0.3178 (3)	0.0804 (8)	
C9	0.70486 (19)	0.40435 (18)	0.08552 (17)	0.0404 (4)	
C10	0.5806 (2)	0.29374 (19)	0.00606 (18)	0.0459 (5)	
H10A	0.4954	0.3003	−0.0021	0.055*	
C11	0.5843 (2)	0.1740 (2)	−0.0606 (2)	0.0531 (5)	
C12	0.7100 (2)	0.1634 (2)	−0.0525 (2)	0.0574 (6)	
H12A	0.7117	0.0828	−0.0982	0.069*	
C13	0.8329 (2)	0.2734 (2)	0.02403 (19)	0.0537 (5)	
C14	0.8311 (2)	0.3936 (2)	0.09427 (18)	0.0474 (5)	
H14A	0.9146	0.4669	0.1472	0.057*	
C15	0.4488 (3)	0.0560 (3)	−0.1458 (3)	0.0801 (8)	
C16	0.9705 (3)	0.2659 (3)	0.0296 (3)	0.0809 (9)	
C17	0.85895 (18)	0.66656 (17)	0.29756 (17)	0.0378 (4)	
C18	0.94351 (19)	0.78052 (18)	0.29999 (18)	0.0422 (4)	
H18A	0.9176	0.7986	0.2337	0.051*	
C19	1.0671 (2)	0.86781 (18)	0.40158 (19)	0.0445 (5)	
C20	1.1054 (2)	0.84267 (19)	0.50081 (19)	0.0475 (5)	
H20A	1.1879	0.9015	0.5688	0.057*	
C21	1.0203 (2)	0.72894 (19)	0.49895 (18)	0.0436 (4)	
C22	0.89764 (19)	0.64089 (18)	0.39795 (18)	0.0417 (4)	
H22A	0.8411	0.5647	0.3970	0.050*	
C23	1.1590 (3)	0.9907 (2)	0.4026 (2)	0.0608 (6)	
C24	1.0639 (3)	0.7057 (2)	0.6091 (2)	0.0627 (6)	
F1A	0.3107 (17)	0.2938 (11)	0.4026 (13)	0.127 (3)	0.64 (3)
F2A	0.3053 (13)	0.1725 (6)	0.2330 (7)	0.106 (2)	0.64 (3)
F3A	0.4911 (12)	0.2685 (12)	0.3845 (8)	0.103 (3)	0.64 (3)
F1B	0.347 (2)	0.3116 (17)	0.4269 (11)	0.101 (4)	0.36 (3)
F2B	0.265 (3)	0.1929 (19)	0.2265 (17)	0.167 (8)	0.36 (3)
F3B	0.474 (3)	0.238 (3)	0.345 (4)	0.143 (8)	0.36 (3)
F4A	0.2145 (11)	0.6369 (9)	0.3520 (13)	0.132 (3)	0.649 (14)
F5A	0.4017 (7)	0.7979 (6)	0.3979 (11)	0.149 (4)	0.649 (14)
F6A	0.2564 (13)	0.6778 (13)	0.2152 (7)	0.149 (4)	0.649 (14)
F4B	0.3833 (18)	0.7925 (14)	0.307 (2)	0.129 (5)	0.351 (14)
F5B	0.2026 (13)	0.6458 (12)	0.242 (2)	0.153 (9)	0.351 (14)
F6B	0.319 (2)	0.733 (2)	0.4278 (10)	0.134 (7)	0.351 (14)
F7A	1.0083 (16)	0.289 (3)	−0.0448 (13)	0.171 (6)	0.52 (2)
F8A	0.9558 (9)	0.1390 (8)	0.010 (2)	0.166 (6)	0.52 (2)
F9A	1.0656 (11)	0.3334 (14)	0.1386 (10)	0.123 (5)	0.52 (2)
F7B	1.0531 (13)	0.3664 (12)	0.009 (2)	0.142 (5)	0.48 (2)
F8B	0.9664 (8)	0.1631 (12)	−0.0554 (10)	0.112 (4)	0.48 (2)
F9B	1.0478 (16)	0.292 (2)	0.1352 (11)	0.141 (6)	0.48 (2)
F10A	0.3748 (10)	0.0774 (10)	−0.2275 (11)	0.124 (4)	0.545 (16)
F11A	0.3666 (10)	0.0405 (10)	−0.0809 (7)	0.124 (3)	0.545 (16)
F12A	0.4594 (10)	−0.0513 (8)	−0.1913 (19)	0.174 (6)	0.545 (16)
F10B	0.4473 (14)	−0.0498 (9)	−0.1405 (11)	0.125 (5)	0.455 (16)
F11B	0.4366 (18)	0.0163 (16)	−0.2650 (6)	0.151 (6)	0.455 (16)
F12B	0.3425 (7)	0.0689 (11)	−0.140 (3)	0.215 (10)	0.455 (16)
F13A	1.2266 (7)	1.0906 (3)	0.5147 (3)	0.107 (2)	0.774 (9)
F14A	1.0835 (3)	1.0359 (4)	0.3402 (5)	0.0925 (13)	0.774 (9)
F15A	1.2459 (5)	0.9734 (4)	0.3515 (6)	0.102 (2)	0.774 (9)
F13B	1.2974 (10)	1.030 (2)	0.481 (2)	0.119 (7)	0.226 (9)
F14B	1.136 (3)	1.0800 (13)	0.438 (4)	0.163 (11)	0.226 (9)
F15B	1.184 (3)	0.9613 (17)	0.3041 (18)	0.156 (11)	0.226 (9)
F16A	1.1855 (12)	0.6770 (8)	0.6200 (8)	0.082 (2)	0.63 (5)
F17A	1.1071 (13)	0.8101 (11)	0.7152 (10)	0.094 (3)	0.63 (5)
F18A	0.9620 (17)	0.6078 (13)	0.6047 (10)	0.092 (3)	0.63 (5)
F16B	1.162 (2)	0.689 (2)	0.625 (2)	0.132 (7)	0.37 (5)
F17B	1.068 (5)	0.802 (2)	0.7088 (18)	0.117 (6)	0.37 (5)
F18B	0.978 (2)	0.5964 (15)	0.6035 (17)	0.073 (4)	0.37 (5)

### Atomic displacement parameters (Å^2^)

**Table:**

	*U*^11^	*U*^22^	*U*^33^	*U*^12^	*U*^13^	*U*^23^
P1	0.0314 (2)	0.0401 (3)	0.0421 (3)	0.0116 (2)	0.01248 (19)	0.0200 (2)
O1	0.0436 (8)	0.0596 (9)	0.0578 (9)	0.0192 (7)	0.0165 (6)	0.0363 (7)
C1	0.0311 (9)	0.0424 (10)	0.0435 (10)	0.0110 (8)	0.0108 (8)	0.0194 (8)
C2	0.0456 (11)	0.0496 (11)	0.0509 (12)	0.0213 (9)	0.0184 (9)	0.0258 (9)
C3	0.0473 (11)	0.0668 (13)	0.0562 (13)	0.0304 (11)	0.0239 (10)	0.0309 (11)
C4	0.0409 (11)	0.0706 (14)	0.0581 (13)	0.0201 (10)	0.0243 (10)	0.0321 (12)
C5	0.0439 (11)	0.0504 (11)	0.0528 (12)	0.0113 (9)	0.0182 (9)	0.0250 (10)
C6	0.0419 (10)	0.0449 (11)	0.0531 (12)	0.0181 (9)	0.0180 (9)	0.0240 (9)
C7	0.0803 (19)	0.0633 (16)	0.084 (2)	0.0198 (15)	0.0439 (17)	0.0412 (16)
C8	0.087 (2)	0.109 (3)	0.098 (2)	0.069 (2)	0.0594 (19)	0.064 (2)
C9	0.0359 (9)	0.0433 (10)	0.0375 (10)	0.0137 (8)	0.0111 (8)	0.0175 (8)
C10	0.0381 (10)	0.0464 (11)	0.0447 (11)	0.0129 (8)	0.0076 (8)	0.0207 (9)
C11	0.0488 (12)	0.0432 (11)	0.0479 (12)	0.0105 (9)	0.0056 (9)	0.0153 (9)
C12	0.0634 (14)	0.0495 (12)	0.0467 (12)	0.0260 (11)	0.0127 (10)	0.0120 (10)
C13	0.0475 (11)	0.0623 (13)	0.0435 (11)	0.0258 (10)	0.0140 (9)	0.0157 (10)
C14	0.0365 (10)	0.0518 (11)	0.0404 (10)	0.0141 (9)	0.0103 (8)	0.0131 (9)
C15	0.0668 (18)	0.0488 (15)	0.079 (2)	0.0073 (13)	−0.0006 (15)	0.0127 (14)
C16	0.0639 (16)	0.093 (2)	0.0667 (18)	0.0435 (17)	0.0220 (15)	0.0102 (16)
C17	0.0345 (9)	0.0346 (9)	0.0435 (10)	0.0132 (7)	0.0170 (8)	0.0164 (8)
C18	0.0431 (10)	0.0394 (10)	0.0453 (11)	0.0158 (8)	0.0205 (8)	0.0191 (8)
C19	0.0430 (10)	0.0332 (9)	0.0508 (11)	0.0101 (8)	0.0226 (9)	0.0137 (9)
C20	0.0388 (10)	0.0391 (10)	0.0475 (11)	0.0075 (8)	0.0123 (9)	0.0115 (9)
C21	0.0408 (10)	0.0410 (10)	0.0428 (11)	0.0144 (8)	0.0122 (8)	0.0168 (8)
C22	0.0382 (10)	0.0351 (9)	0.0477 (11)	0.0101 (8)	0.0158 (8)	0.0187 (8)
C23	0.0576 (14)	0.0456 (13)	0.0674 (15)	0.0077 (11)	0.0273 (12)	0.0216 (12)
C24	0.0612 (16)	0.0544 (14)	0.0514 (14)	0.0100 (12)	0.0050 (11)	0.0234 (12)
F1A	0.156 (6)	0.100 (4)	0.199 (9)	0.056 (5)	0.140 (7)	0.095 (5)
F2A	0.147 (6)	0.0449 (19)	0.087 (3)	0.008 (3)	0.025 (3)	0.028 (2)
F3A	0.122 (5)	0.106 (4)	0.112 (4)	0.048 (4)	0.041 (4)	0.082 (3)
F1B	0.126 (8)	0.108 (6)	0.062 (6)	0.012 (5)	0.039 (5)	0.059 (4)
F2B	0.131 (9)	0.079 (7)	0.196 (14)	−0.035 (6)	−0.045 (8)	0.094 (9)
F3B	0.172 (17)	0.151 (14)	0.28 (2)	0.125 (13)	0.168 (16)	0.178 (16)
F4A	0.153 (6)	0.162 (5)	0.219 (9)	0.124 (5)	0.154 (6)	0.131 (6)
F5A	0.138 (4)	0.082 (3)	0.185 (8)	0.075 (3)	0.027 (5)	0.016 (4)
F6A	0.191 (8)	0.279 (10)	0.141 (4)	0.204 (8)	0.107 (6)	0.152 (6)
F4B	0.152 (11)	0.130 (8)	0.209 (14)	0.112 (8)	0.119 (11)	0.109 (9)
F5B	0.077 (6)	0.133 (7)	0.193 (15)	0.075 (6)	−0.001 (6)	0.025 (8)
F6B	0.189 (15)	0.206 (15)	0.096 (6)	0.162 (14)	0.088 (8)	0.075 (8)
F7A	0.129 (8)	0.337 (18)	0.133 (7)	0.135 (11)	0.106 (7)	0.128 (10)
F8A	0.103 (4)	0.117 (5)	0.245 (14)	0.081 (4)	0.045 (7)	0.035 (7)
F9A	0.044 (3)	0.141 (5)	0.100 (7)	0.043 (3)	0.000 (3)	−0.015 (5)
F7B	0.089 (5)	0.136 (7)	0.263 (15)	0.059 (4)	0.118 (8)	0.111 (7)
F8B	0.087 (3)	0.101 (5)	0.108 (6)	0.054 (4)	0.041 (3)	−0.008 (4)
F9B	0.094 (8)	0.283 (17)	0.092 (8)	0.119 (10)	0.042 (6)	0.098 (10)
F10A	0.097 (5)	0.083 (4)	0.093 (5)	−0.011 (3)	−0.048 (4)	0.029 (4)
F11A	0.076 (5)	0.111 (5)	0.111 (4)	−0.028 (3)	0.020 (3)	0.037 (3)
F12A	0.093 (4)	0.052 (4)	0.230 (12)	0.018 (3)	0.008 (7)	−0.044 (5)
F10B	0.131 (8)	0.055 (5)	0.108 (5)	−0.022 (5)	−0.014 (5)	0.037 (5)
F11B	0.157 (9)	0.101 (6)	0.063 (3)	−0.033 (6)	−0.031 (4)	0.014 (4)
F12B	0.042 (3)	0.084 (7)	0.32 (2)	0.004 (3)	0.003 (9)	−0.052 (10)
F13A	0.117 (4)	0.0482 (17)	0.0828 (18)	−0.024 (2)	0.0195 (18)	0.0112 (12)
F14A	0.0812 (16)	0.061 (2)	0.141 (3)	0.0181 (15)	0.033 (2)	0.065 (2)
F15A	0.096 (3)	0.075 (2)	0.185 (6)	0.042 (2)	0.105 (4)	0.071 (3)
F13B	0.050 (5)	0.114 (11)	0.152 (12)	−0.021 (6)	0.003 (6)	0.084 (10)
F14B	0.181 (19)	0.039 (7)	0.34 (3)	0.065 (11)	0.207 (19)	0.072 (13)
F15B	0.189 (18)	0.103 (10)	0.097 (9)	−0.053 (12)	0.077 (12)	0.036 (6)
F16A	0.054 (3)	0.098 (5)	0.101 (4)	0.036 (3)	0.010 (2)	0.063 (4)
F17A	0.111 (5)	0.066 (4)	0.045 (3)	0.003 (5)	−0.007 (3)	0.013 (2)
F18A	0.078 (4)	0.111 (6)	0.065 (4)	−0.003 (4)	0.012 (2)	0.059 (4)
F16B	0.093 (7)	0.223 (18)	0.171 (12)	0.108 (10)	0.048 (7)	0.149 (13)
F17B	0.205 (18)	0.109 (11)	0.039 (6)	0.080 (10)	0.038 (8)	0.028 (6)
F18B	0.082 (7)	0.052 (5)	0.070 (6)	0.018 (5)	0.005 (4)	0.035 (4)

### Geometric parameters (Å, °)

**Table:**

P1—O1	1.4726 (15)	C14—H14A	0.9300
P1—C9	1.8122 (19)	C15—F12B	1.215 (9)
P1—C17	1.8160 (19)	C15—F12A	1.246 (7)
P1—C1	1.8163 (19)	C15—F10B	1.277 (9)
C1—C6	1.386 (3)	C15—F10A	1.290 (7)
C1—C2	1.387 (3)	C15—F11B	1.352 (10)
C2—C3	1.386 (3)	C15—F11A	1.354 (8)
C2—H2A	0.9300	C16—F7A	1.187 (11)
C3—C4	1.376 (3)	C16—F9B	1.256 (11)
C3—C8	1.500 (3)	C16—F8B	1.279 (7)
C4—C5	1.379 (3)	C16—F9A	1.280 (10)
C4—H4A	0.9300	C16—F8A	1.392 (11)
C5—C6	1.392 (3)	C16—F7B	1.417 (9)
C5—C7	1.498 (3)	C17—C18	1.385 (3)
C6—H6A	0.9300	C17—C22	1.394 (3)
C7—F3B	1.286 (19)	C18—C19	1.391 (3)
C7—F2A	1.287 (7)	C18—H18A	0.9300
C7—F1B	1.320 (13)	C19—C20	1.377 (3)
C7—F1A	1.321 (8)	C19—C23	1.506 (3)
C7—F3A	1.323 (11)	C20—C21	1.389 (3)
C7—F2B	1.368 (15)	C20—H20A	0.9300
C8—F5B	1.232 (10)	C21—C22	1.383 (3)
C8—F5A	1.263 (6)	C21—C24	1.496 (3)
C8—F6A	1.285 (7)	C22—H22A	0.9300
C8—F6B	1.293 (8)	C23—F14B	1.117 (9)
C8—F4A	1.341 (5)	C23—F15B	1.268 (16)
C8—F4B	1.374 (10)	C23—F15A	1.277 (4)
C9—C14	1.387 (3)	C23—F13A	1.317 (4)
C9—C10	1.393 (3)	C23—F14A	1.346 (4)
C10—C11	1.384 (3)	C23—F13B	1.412 (10)
C10—H10A	0.9300	C24—F16B	1.11 (3)
C11—C12	1.382 (3)	C24—F17A	1.308 (11)
C11—C15	1.504 (3)	C24—F18A	1.327 (11)
C12—C13	1.377 (3)	C24—F18B	1.330 (17)
C12—H12A	0.9300	C24—F17B	1.33 (2)
C13—C14	1.390 (3)	C24—F16A	1.436 (15)
C13—C16	1.500 (3)		
			
O1—P1—C9	114.30 (9)	F10B—C15—F11B	97.3 (8)
O1—P1—C17	111.18 (8)	F10A—C15—F11B	52.6 (6)
C9—P1—C17	107.20 (8)	F12A—C15—F11A	105.5 (8)
O1—P1—C1	113.04 (8)	F10B—C15—F11A	77.7 (7)
C9—P1—C1	105.36 (9)	F10A—C15—F11A	101.5 (6)
C17—P1—C1	105.12 (8)	F11B—C15—F11A	139.0 (6)
C6—C1—C2	119.39 (18)	F12B—C15—C11	116.7 (4)
C6—C1—P1	123.91 (14)	F12A—C15—C11	115.2 (5)
C2—C1—P1	116.70 (15)	F10B—C15—C11	113.1 (6)
C3—C2—C1	120.0 (2)	F10A—C15—C11	111.6 (4)
C3—C2—H2A	120.0	F11B—C15—C11	110.5 (4)
C1—C2—H2A	120.0	F11A—C15—C11	108.7 (4)
C4—C3—C2	120.7 (2)	F7A—C16—F9B	126.0 (9)
C4—C3—C8	120.1 (2)	F7A—C16—F8B	67.9 (8)
C2—C3—C8	119.2 (2)	F9B—C16—F8B	112.0 (8)
C3—C4—C5	119.53 (19)	F7A—C16—F9A	112.2 (10)
C3—C4—H4A	120.2	F8B—C16—F9A	124.6 (7)
C5—C4—H4A	120.2	F7A—C16—F8A	109.7 (7)
C4—C5—C6	120.4 (2)	F9B—C16—F8A	80.4 (9)
C4—C5—C7	120.3 (2)	F9A—C16—F8A	100.0 (9)
C6—C5—C7	119.3 (2)	F9B—C16—F7B	101.4 (8)
C1—C6—C5	120.01 (18)	F8B—C16—F7B	100.6 (6)
C1—C6—H6A	120.0	F9A—C16—F7B	82.5 (9)
C5—C6—H6A	120.0	F8A—C16—F7B	136.6 (8)
F3B—C7—F2A	86.2 (14)	F7A—C16—C13	112.9 (6)
F3B—C7—F1B	108.0 (14)	F9B—C16—C13	113.6 (7)
F2A—C7—F1B	118.1 (8)	F8B—C16—C13	116.5 (4)
F3B—C7—F1A	118.5 (11)	F9A—C16—C13	113.4 (6)
F2A—C7—F1A	104.9 (7)	F8A—C16—C13	107.7 (6)
F2A—C7—F3A	104.5 (7)	F7B—C16—C13	110.8 (5)
F1B—C7—F3A	92.2 (9)	C18—C17—C22	119.66 (17)
F1A—C7—F3A	105.4 (8)	C18—C17—P1	117.27 (15)
F3B—C7—F2B	110.3 (12)	C22—C17—P1	122.98 (14)
F1B—C7—F2B	108.4 (11)	C17—C18—C19	119.98 (19)
F1A—C7—F2B	92.6 (10)	C17—C18—H18A	120.0
F3A—C7—F2B	128.3 (13)	C19—C18—H18A	120.0
F3B—C7—C5	114.7 (9)	C20—C19—C18	120.43 (18)
F2A—C7—C5	115.9 (5)	C20—C19—C23	120.03 (18)
F1B—C7—C5	111.4 (7)	C18—C19—C23	119.5 (2)
F1A—C7—C5	113.4 (4)	C19—C20—C21	119.68 (18)
F3A—C7—C5	111.8 (6)	C19—C20—H20A	120.2
F2B—C7—C5	103.9 (8)	C21—C20—H20A	120.2
F5B—C8—F5A	126.4 (6)	C22—C21—C20	120.38 (19)
F5A—C8—F6A	110.9 (6)	C22—C21—C24	121.32 (18)
F5B—C8—F6B	108.5 (8)	C20—C21—C24	118.29 (18)
F5A—C8—F6B	57.5 (8)	C21—C22—C17	119.86 (17)
F6A—C8—F6B	133.5 (5)	C21—C22—H22A	120.1
F5B—C8—F4A	65.5 (10)	C17—C22—H22A	120.1
F5A—C8—F4A	109.6 (5)	F14B—C23—F15B	117.7 (15)
F6A—C8—F4A	102.7 (5)	F14B—C23—F15A	128.5 (7)
F6B—C8—F4A	54.4 (8)	F14B—C23—F13A	55 (2)
F5B—C8—F4B	96.6 (8)	F15B—C23—F13A	133.0 (8)
F5A—C8—F4B	48.0 (6)	F15A—C23—F13A	109.2 (3)
F6A—C8—F4B	66.4 (7)	F14B—C23—F14A	50 (2)
F6B—C8—F4B	101.1 (9)	F15B—C23—F14A	76.9 (14)
F4A—C8—F4B	136.2 (4)	F15A—C23—F14A	105.2 (3)
F5B—C8—C3	119.3 (6)	F13A—C23—F14A	103.7 (3)
F5A—C8—C3	112.2 (3)	F14B—C23—F13B	104.7 (14)
F6A—C8—C3	110.6 (4)	F15B—C23—F13B	95.3 (11)
F6B—C8—C3	115.2 (4)	F15A—C23—F13B	62.9 (10)
F4A—C8—C3	110.3 (3)	F13A—C23—F13B	53.5 (10)
F4B—C8—C3	113.1 (4)	F14A—C23—F13B	139.5 (5)
C14—C9—C10	119.36 (18)	F14B—C23—C19	117.4 (6)
C14—C9—P1	122.02 (14)	F15B—C23—C19	110.3 (7)
C10—C9—P1	118.42 (14)	F15A—C23—C19	113.7 (3)
C11—C10—C9	119.79 (18)	F13A—C23—C19	112.7 (2)
C11—C10—H10A	120.1	F14A—C23—C19	111.5 (2)
C9—C10—H10A	120.1	F13B—C23—C19	108.5 (5)
C12—C11—C10	120.84 (18)	F16B—C24—F17A	97.2 (13)
C12—C11—C15	120.3 (2)	F16B—C24—F18A	110.4 (12)
C10—C11—C15	118.9 (2)	F17A—C24—F18A	108.7 (7)
C13—C12—C11	119.3 (2)	F16B—C24—F18B	99.9 (13)
C13—C12—H12A	120.3	F17A—C24—F18B	113.2 (9)
C11—C12—H12A	120.3	F16B—C24—F17B	112.9 (19)
C12—C13—C14	120.58 (19)	F18A—C24—F17B	96.7 (14)
C12—C13—C16	120.3 (2)	F18B—C24—F17B	103.4 (16)
C14—C13—C16	119.1 (2)	F17A—C24—F16A	101.4 (7)
C9—C14—C13	120.04 (18)	F18A—C24—F16A	109.1 (9)
C9—C14—H14A	120.0	F18B—C24—F16A	98.5 (9)
C13—C14—H14A	120.0	F17B—C24—F16A	117 (2)
F12B—C15—F12A	125.6 (6)	F16B—C24—C21	114.0 (12)
F12B—C15—F10B	110.1 (12)	F17A—C24—C21	114.0 (7)
F12B—C15—F10A	59.9 (12)	F18A—C24—C21	111.6 (6)
F12A—C15—F10A	113.0 (7)	F18B—C24—C21	116.0 (8)
F10B—C15—F10A	132.8 (6)	F17B—C24—C21	109.9 (12)
F12B—C15—F11B	107.1 (10)	F16A—C24—C21	111.6 (4)
F12A—C15—F11B	67.5 (6)		
			
O1—P1—C1—C6	150.78 (16)	C10—C11—C15—F12A	−171.8 (13)
C9—P1—C1—C6	25.28 (19)	C12—C11—C15—F10B	44.2 (9)
C17—P1—C1—C6	−87.79 (17)	C10—C11—C15—F10B	−137.9 (8)
O1—P1—C1—C2	−28.91 (18)	C12—C11—C15—F10A	−120.4 (10)
C9—P1—C1—C2	−154.40 (15)	C10—C11—C15—F10A	57.5 (10)
C17—P1—C1—C2	92.53 (16)	C12—C11—C15—F11B	−63.8 (13)
C6—C1—C2—C3	−0.2 (3)	C10—C11—C15—F11B	114.2 (13)
P1—C1—C2—C3	179.48 (16)	C12—C11—C15—F11A	128.4 (6)
C1—C2—C3—C4	−0.5 (3)	C10—C11—C15—F11A	−53.6 (7)
C1—C2—C3—C8	−179.6 (2)	C12—C13—C16—F7A	88.9 (15)
C2—C3—C4—C5	0.7 (3)	C14—C13—C16—F7A	−89.2 (15)
C8—C3—C4—C5	179.8 (2)	C12—C13—C16—F9B	−119.3 (12)
C3—C4—C5—C6	−0.3 (3)	C14—C13—C16—F9B	62.5 (12)
C3—C4—C5—C7	−177.3 (2)	C12—C13—C16—F8B	13.2 (11)
C2—C1—C6—C5	0.7 (3)	C14—C13—C16—F8B	−165.0 (10)
P1—C1—C6—C5	−179.01 (15)	C12—C13—C16—F9A	−142.0 (9)
C4—C5—C6—C1	−0.4 (3)	C14—C13—C16—F9A	39.9 (9)
C7—C5—C6—C1	176.6 (2)	C12—C13—C16—F8A	−32.3 (13)
C4—C5—C7—F3B	−160 (2)	C14—C13—C16—F8A	149.6 (13)
C6—C5—C7—F3B	23 (2)	C12—C13—C16—F7B	127.3 (10)
C4—C5—C7—F2A	101.8 (7)	C14—C13—C16—F7B	−50.9 (10)
C6—C5—C7—F2A	−75.3 (7)	O1—P1—C17—C18	−8.74 (17)
C4—C5—C7—F1B	−37.0 (9)	C9—P1—C17—C18	116.84 (15)
C6—C5—C7—F1B	145.9 (9)	C1—P1—C17—C18	−131.39 (14)
C4—C5—C7—F1A	−19.6 (9)	O1—P1—C17—C22	167.85 (15)
C6—C5—C7—F1A	163.3 (9)	C9—P1—C17—C22	−66.56 (17)
C4—C5—C7—F3A	−138.6 (5)	C1—P1—C17—C22	45.21 (17)
C6—C5—C7—F3A	44.3 (6)	C22—C17—C18—C19	0.8 (3)
C4—C5—C7—F2B	79.5 (15)	P1—C17—C18—C19	177.48 (14)
C6—C5—C7—F2B	−97.6 (15)	C17—C18—C19—C20	−0.8 (3)
C4—C3—C8—F5B	−78.3 (16)	C17—C18—C19—C23	179.34 (18)
C2—C3—C8—F5B	100.9 (16)	C18—C19—C20—C21	0.3 (3)
C4—C3—C8—F5A	116.9 (9)	C23—C19—C20—C21	−179.81 (19)
C2—C3—C8—F5A	−64.0 (9)	C19—C20—C21—C22	0.2 (3)
C4—C3—C8—F6A	−118.7 (8)	C19—C20—C21—C24	−179.0 (2)
C2—C3—C8—F6A	60.5 (8)	C20—C21—C22—C17	−0.2 (3)
C4—C3—C8—F6B	53.5 (15)	C24—C21—C22—C17	178.99 (19)
C2—C3—C8—F6B	−127.4 (15)	C18—C17—C22—C21	−0.3 (3)
C4—C3—C8—F4A	−5.7 (8)	P1—C17—C22—C21	−176.79 (14)
C2—C3—C8—F4A	173.4 (7)	C20—C19—C23—F14B	−93 (3)
C4—C3—C8—F4B	169.1 (12)	C18—C19—C23—F14B	87 (3)
C2—C3—C8—F4B	−11.7 (13)	C20—C19—C23—F15B	128.5 (17)
O1—P1—C9—C14	98.19 (17)	C18—C19—C23—F15B	−51.6 (17)
C17—P1—C9—C14	−25.50 (19)	C20—C19—C23—F15A	93.1 (4)
C1—P1—C9—C14	−137.10 (17)	C18—C19—C23—F15A	−87.0 (4)
O1—P1—C9—C10	−76.64 (17)	C20—C19—C23—F13A	−31.9 (5)
C17—P1—C9—C10	159.67 (16)	C18—C19—C23—F13A	148.0 (4)
C1—P1—C9—C10	48.06 (18)	C20—C19—C23—F14A	−148.1 (3)
C14—C9—C10—C11	1.7 (3)	C18—C19—C23—F14A	31.8 (4)
P1—C9—C10—C11	176.64 (16)	C20—C19—C23—F13B	25.4 (14)
C9—C10—C11—C12	−2.1 (3)	C18—C19—C23—F13B	−154.8 (14)
C9—C10—C11—C15	−180.0 (2)	C22—C21—C24—F16B	115.3 (11)
C10—C11—C12—C13	0.7 (4)	C20—C21—C24—F16B	−65.5 (12)
C15—C11—C12—C13	178.5 (2)	C22—C21—C24—F17A	−134.3 (7)
C11—C12—C13—C14	1.1 (4)	C20—C21—C24—F17A	44.9 (7)
C11—C12—C13—C16	−177.0 (3)	C22—C21—C24—F18A	−10.7 (9)
C10—C9—C14—C13	0.1 (3)	C20—C21—C24—F18A	168.5 (8)
P1—C9—C14—C13	−174.68 (17)	C22—C21—C24—F18B	0.0 (9)
C12—C13—C14—C9	−1.5 (3)	C20—C21—C24—F18B	179.2 (9)
C16—C13—C14—C9	176.6 (2)	C22—C21—C24—F17B	−117 (2)
C12—C11—C15—F12B	173 (2)	C20—C21—C24—F17B	62 (2)
C10—C11—C15—F12B	−9(2)	C22—C21—C24—F16A	111.6 (3)
C12—C11—C15—F12A	10.3 (14)	C20—C21—C24—F16A	−69.2 (4)

### Hydrogen-bond geometry (Å, °)

**Table:**

*D*—H···*A*	*D*—H	H···*A*	*D*···*A*	*D*—H···*A*
C18—H18A···O1	0.93	2.58	2.992 (3)	108
C10—H10A···O1^i^	0.93	2.38	3.203 (3)	147
C20—H20A···F10A^ii^	0.93	2.50	3.418 (12)	167

Symmetry codes: (i) −*x*+1, −*y*+1, −*z*; (ii) *x*+1, *y*+1, *z*+1.
